# Audiology practice management in South Africa: What audiologists know and what they should know

**DOI:** 10.4102/sajcd.v62i1.114

**Published:** 2015-12-09

**Authors:** Deidré Breytenbach, Alta Kritzinger, Maggi Soer

**Affiliations:** 1 Department of Speech-Language Pathology and Audiology, University of Pretoria, South Africa

## Abstract

**Background:**

In future, the South African Department of Health aims to purchase services from accredited private service providers. Successful private audiology practices can assist to address issues of access, equity and quality of health services. It is not sufficient to be an excellent clinician, since audiology practices are businesses that must also be managed effectively.

**Objective:**

The objective was to determine the existing and required levels of practice management knowledge as perceived by South African audiologists.

**Method:**

An electronic descriptive survey was used to investigate audiology practice management amongst South African audiologists. A total of 147 respondents completed the survey. Results were analysed by calculating descriptive statistics. The Z-proportional test was used to identify significant differences between existing and required levels of practice management knowledge.

**Results:**

Significant differences were found between existing and required levels of knowledge regarding all eight practice management tasks, particularly legal and ethical issues and marketing and accounting. There were small differences in the knowledge required for practice management tasks amongst respondents working in public and private settings.

**Conclusion:**

Irrespective of their work context, respondents showed that they need significant expansion of practice management knowledge in order to be successful, to compete effectively and to make sense of a complex marketplace.

## Introduction

Audiology in South Africa has, over the past five decades, advanced from a combined profession of speech and hearing therapy into two interweaved, but autonomous, professions of audiology and speech-language therapy (Edwards, 2009; Swanepoel, 2006). Audiology has now diversified within this multiracial, multilingual, and multicultural context as a hearing healthcare profession aimed at providing quality services to meet the diverse needs of the entire population (Swanepoel, 2006). Audiologists are offered a wide selection of practice opportunities across a number of work settings in South Africa. The country has a large public sector and a smaller but fast-growing private healthcare sector (Bakker, 2008). According to Lefemine (2012), the private sector in South Africa attracts the majority of the country's health professionals. Consequently, there is a shortage and maldistribution of key healthcare workers across the rural-urban and public-private divides (Swanepoel, 2006; Ward, Sanders, Leng & Pollock, 2014). Annually, an estimated 6116 infants will be born with or acquire permanent bilateral hearing loss, with approximately 92% born in the public health sector (Swanepoel, Storbeck & Friedland, 2009). Therefore, one of the main challenges in the public health care sector is a shortage of audiologists (Kanji & Kara, 2013). As part of improving the healthcare system and ensuring that all South Africans have equitable access to essential healthcare services, the South African Government is introducing the National Health Insurance (NHI) system. In future, the Department of Health aims to purchase services from accredited private service providers (Matsoso & Fryatt, 2013; Ward *et al*., 2014). The strategy aims to increase the number of health care personnel indirectly by enjoining those in the private sector to provide services to the general public (George, Quinlan, Reardron & Aguilera, 2012). The success of private audiology practices is, therefore, also important for the future engagement between sectors in order to address issues of access, equity and quality of health services by increasing private sector participation.

The future of private audiology practices depends on how well audiologists are able to take their clinical training and practice management skills to the marketplace (Metz, 1996). This basic and longstanding requirement also applies to the future of audiology practices in South Africa. A successful audiologist must provide exceptional patient care and customer service regardless of the setting, but if equal attention is not paid to the business aspect of the practice the success of the practice could be risked (Gnewikow, Gnewikow & Cieliczka, 2009; Hosford-Dunn, Roeser & Valente, 2008). According to Clark and Benson (2008) audiologists must understand the difficulty of balancing the need to serve the public in the highest ethical manner and also making a living by operating a business. The practice management requirements of the private audiology practice may also differ from the audiologist's patient-first professional motives (Hosford-Dunn *et al*., 2008). Therefore, practice management is the most underestimated challenge to the private audiologist, since every audiology practice becomes a business that must also be managed effectively (Gnewikow *et al*., 2009; Traynor, 2006). Although a wide variety of core audiology skills need to be mastered prior to working as an audiologist, practice management proficiency is of equal importance in any environment. Audiologists must be able to use many management skills in practice that may not have been acquired in undergraduate training (Clark & Benson, 2008).

There appears to be a lack of recent research regarding practice management needs amongst South African audiologists. In a country-wide South African survey by Wemmer (2007) a significant number of respondents (35%) indicated that their undergraduate education left them unprepared for practice management, whilst 22% indicated that it had not been included in the undergraduate curriculum. In another South African study conducted by Bakker (2008), 86% of respondents did not receive practice management training in their undergraduate studies. According to a survey of audiologists (*n* = 256) in the United States of America (USA) and Canada (Henson, Williamson & Jacques, 2006) it was clear that practicing audiologists in those countries also felt they did not have the required business skills to compete in the marketplace. Respondents reported great deficits in management and business knowledge that impacted their work and careers (Henson *et al*., 2006). It is not currently known how practice management training is perceived by audiologists in South Africa. It was therefore important to conduct a survey to determine context specific management challenges, strengths and training needs as well as the self-perceived existing and required levels of practice management knowledge amongst South African audiologists. This survey included eight practice management tasks that may be considered as the most important body of knowledge for business, namely accounting, finance, marketing, legal and ethical issues, organisational behaviour and human resources, operations and systems management, strategic management, and managerial decision-making (Henson *et al*., 2006; Henson, Presley & Korfmann, 2008; Hosford-Dunn *et al*., 2008). The results may identify differences between the existing levels of knowledge and the required levels of knowledge of the respondents. This may lead to the formulation of recommendations regarding practice management training for South African audiologists.

## Method

### Aim

The aim of the study was to determine the self-perceived existing and required levels of practice management knowledge amongst South African audiologists.

### Objectives

- To determine the existing levels of knowledge necessary to perform practice management tasks as perceived by the respondents.
- To determine the required levels of knowledge necessary to perform practice management tasks as perceived by the respondents.
- To determine if there are differences between the existing levels of knowledge and the required levels of knowledge necessary to perform practice management tasks as perceived by the respondents.
- To determine if there are differences between the existing levels of knowledge and the required levels of knowledge between respondents working in public settings and private settings.
- To describe strengths, management challenges and training needs as perceived by the respondents.
- To describe the respondents’ recommendations regarding practice management training for South African audiologists.

### Research design

A descriptive survey using electronic questionnaire distribution was used to investigate audiology practice management amongst South African audiologists. A web-based survey was deemed the most effective method to gather the opinions of as many South African audiologists as possible.

### Ethical considerations

Ethical clearance to conduct the study was obtained from the Research Ethics Committee of the Faculty of Humanities at the University of Pretoria (Reference number: 26162289). The data was collected anonymously and treated with confidentiality. No identifying information of the respondents was reported and internet protocol addresses were not tracked. An informed consent letter formed part of the web-based survey and was the first page that respondents viewed once they clicked on the link. By completing the web-based survey respondents gave their consent to participate in the study on a voluntary basis.

### Respondents

The population of interest was registered audiologists and dually qualified speech-language therapists and audiologists working in public or private settings in South Africa – whether they are involved in practice management or not. A non-probability, convenience sampling strategy was employed. Because a large number of audiologists are still dually qualified, it was not possible to determine how many of the professionals are practicing only as audiologists. At the time of data collection there were 1749 audiologists and dually registered speech-language therapists and audiologists registered with the Health Professions Council of South Africa (HPCSA, 2013), but email addresses could not be obtained. The HPCSA does not supply practitioner contact information such as email addresses, but postal addresses may be purchased from them (HPCSA, 2013). In addition to their professional registration, some audiologists also joined professional organisations such as the South African Association of Audiologists (SAAA) and the South African Speech-Language-Hearing Association (SASLHA). The email was sent to the databases of SAAA (*n* = 326) and SASLHA (*n* = 1300) as their member databases are updated annually, and they were able to send the survey to all their members. Since there was no effective way to determine which of the SASLHA members practice only as speech-language therapists, a number of redundant emails were sent to speech-language therapists. Some audiologists are also registered with both the SAAA and SASLHA, meaning that some respondents might have received an invitation to participate twice. An email message providing the web address where the respondent could link directly to the survey was thus sent to a total of approximately 1626 prospective respondents. A total of 147 respondents completed the survey indicating a minimum response rate of 9%. A description of the respondents is given in Table 1.

**TABLE 1:** Description of Respondents (*n*=147).

Characteristics	Values	*f*(n)	%
Gender distribution (six missing values)	Male	3	2.1
	Female	138	97.9
Age	Mean age (years)	34.9	–
	Median age	32	–
	Minimum	23	–
	Maximum	61	–
	Standard deviation	9.63	–
Employment status (six missing values)	Employer/owner	45	31.9
	Practice manager	17	12.1
	Full-time employee	59	41.8
	Part-time employee	10	7.1
	Other	10	7.1
Current employment context (six missing values)	Government hospital	25	17.7
	Private hospital	3	2
	Private practice: own venture	39	27.7
	Private practice: together with/under another person	38	27
	School setting	9	6.4
	Academic setting	7	5.0
	Hearing aid company	12	8.5
	Other	8	5.7
Institution where undergraduate training was completed (11 missing values)	University of Cape Town	13	9.6
	University of KwaZulu-Natal (Durban Westville)	16	11.8
	University of Limpopo (Medunsa)	3	2.2
	University of Stellenbosch	8	5.9
	University of Pretoria	75	55.1
	University of the Witwatersrand	20	14.7
	Other	1	0.7
Professional qualifications (11 missing values)	Speech-language Therapist and Audiologist (STA)	89	65.4
	Audiologist (AU)	47	34.6
Academic qualification (11 missing values)	Bachelor in Speech-language Pathology and Audiology	72	52.9
	Bachelor in Audiology	30	22.1
	Masters in Audiology	17	12.5
	Doctorate in Audiology (DPhil)	1	0.7
	Doctorate in Audiology (AuD)	2	1.5
	MBA	4	2.9
	Other qualifications not related to practice management	10	7.4
Practice location (11 missing values)	Urban	101	74.3
	Semi-urban	25	18.4
	Rural	10	7.4
Province where employed (six missing values)	The Eastern Cape	2	1.4
	The Free State	5	3.5
	Gauteng	67	47.5
	KwaZulu-Natal	20	14.2
	Limpopo	9	6.4
	Mpumalanga	7	5.0
	The Northern Cape	1	0.7
	The North West	5	3.5
	The Western Cape	25	17.7

According to Table 1 the respondents consisted mostly of females (97.9%), which is consistent with the population of audiologists in South Africa (Wemmer, 2007). Ages ranged between 23 and 61 years with a mean age of 34.9 years. The majority of respondents were dually qualified as speech-language therapists and audiologists (65.4%) with a bachelor's degree obtained from the University of Pretoria (55.1%). The small sample size and the fact that most respondents graduated from one tertiary institution influenced the generalisability of the findings, especially findings regarding training. The largest group of respondents were full time employees (41.8%) working in a private practice (54.6%) located in an urban area (74.3%) in the Gauteng province (47.5%). Very few respondents worked in a public setting (30.8%) compared to those who worked in a private context (69.2%). It is possible that private practitioners had a greater interest in the topic of practice management than respondents working in a public setting, and therefore more respondents from a private context participated in the study.

There is also incongruity regarding the number of respondents employed in each province and the size of each province. Some provinces, such as the Northern Cape, were underrepresented in the survey. There were a few missing values (*n* = 11) as demographic information was obtained last in the web-based survey, and some respondents did not complete all the questions.

### Materials

A web-based survey was developed by consulting previous studies regarding practice management (Henson *et al*., 2006), and refined after a pilot study was completed. Structured closed-ended questions and open-ended questions were used in the survey. Section A was assigned to training and education in practice management to gather information regarding the level of training respondents have received in practice management. This section requested the respondent's opinions regarding the need for practice management training, and when and how it should be presented. Their opinions on what the content, duration, and format of such training should entail was also requested. Section B was assigned to management in practice. This section was used to determine the respondents’ challenges as well as strengths in practice management. In this section the researcher also determined the existing and required levels of knowledge necessary to perform practice management tasks as perceived by the respondents. Questions in this section were based on a study conducted by Henson *et al*. (2006), which included eight areas which may be considered as the most essential knowledge for business, namely accounting, finance, marketing, legal and ethical issues, organisational behaviour and human resources, operations and systems management, strategic management, and managerial decision-making. Section C was assigned to demographic information to gather information about the profile of respondents and their audiology practices. According to Haslam and McGarty (2014), demographic information should be gathered at the end of a survey as respondents tend to lose interest when too much demographic information is asked in the beginning.

### Procedures

A pilot study was conducted to pre-test the web-based survey. The preliminary survey was sent to nine respondents. They recommended changes to the flow of the questions and the format of skip-questions to enhance the efficacy and practicality of the survey. For the main study, SAAA and SASLHA sent an email message to their databases. The email message consisted of a cover letter addressed to prospective respondents, inviting them to participate in an anonymous web-based survey. The message also provided the web address to link directly to the survey. SurveyMonkey was used to collect the data online. Two weeks after the first email was sent, a second email was sent reminding prospective respondents to complete the survey and informing them of the closing date. The survey was open for participation for three months as data collection coincided with December 2013 and January 2014 school holidays.

### Data analysis

The data from SurveyMonkey was exported into the Statistical Package for the Social Sciences (*IBM SPSS, Version 22*) for statistical analysis. Results were quantitatively analysed by calculating descriptive statistics such as percentages, frequency distribution, measures of central tendency and standard deviation. This assisted in organising and summarising the data. The Z-proportional test (Maree, 2007) was used to determine if two groups of respondents differed significantly on selected characteristics. Content analysis (Leedy & Ormrod, 2010) was also used to analyse qualitative data derived from open-ended questions. Underlying patterns, key themes and trends were identified by thorough and systematic examination of the text data collected from the web-based survey.

## Results and discussion

### Existing knowledge of audiology practice management tasks

Respondents were requested to evaluate their perceived existing levels of knowledge regarding eight practice management tasks and rate them as very low, low, high or very high on a 4-point Likert scale. Very low and low results and high and very high results were combined to summarise the data. The results are presented in Table 2.

**TABLE 2:** Existing levels of knowledge (n=147).

Management task	Low and very low knowledge (%)	High and very high knowledge (%)	Ranking according to high and very high knowledge
1. Accounting	62.6	37.4	8
2. Finance	57.1	42.9	5
3. Marketing	56.5	43.5	3
4. Legal and ethical issues	60.6	39.4	7
5. Organisational behaviour and human resources	55.1	44.9	2
6. Operations and systems management (one missing value)	43.8	56.1	1
7. Strategic management	56.5	43.5	4
8. Managerial decision-making	59.2	40.8	6

Results in Table 2 indicated that respondents’ existing knowledge of practice management tasks were mostly low or very low for all eight practice management tasks. Operations and systems management was the practice management task respondents reported they knew the most about (56.1%). Operations and systems management is an integral part of an audiologist's daily tasks, which includes managing the processes that produce and distribute products and services such as diagnostic hearing tests and the fitting of hearing aids. Respondents may therefore mostly report a high level of existing knowledge regarding operations and systems management. Accounting was the task they knew the least about (37.4%). The results were in agreement with Henson *et al*. (2008), who found that accounting was the task that chiropractors in the USA knew the least about. Knowledge of accounting is necessary to make financial decisions – from purchasing equipment and supplies to expanding services and determining salaries (Traynor, 2008). Accounting is a specialised field and would not have been included during undergraduate studies. According to Clark and Benson (2008) audiologists must have an understanding of basic bookkeeping and accounting.

### Required knowledge to perform audiology practice management tasks

Respondents were requested to indicate required levels of knowledge regarding eight practice management tasks and rate them as very low, low, high or very high on a 4-point Likert scale. Very low and low results and high or very high results were combined to summarise the data. The results are presented in Table 3.

**TABLE 3:** Required levels of knowledge (*n=*147).

Management task	Very low and low required knowledge (%)	High and very high required knowledge (%)	Ranking according to high and very high required levels of knowledge
1. Accounting (two missing values)	8.3	91.7	5
2. Finance	8.2	91.8	3
3. Marketing (three missing values)	4.2	95.8	1
4. Legal and ethical issues	4.8	95.2	2
5. Organisational behaviour and human resources	10.9	89.1	8
6. Operations and systems management (one missing value)	10.3	89.7	6
7. Strategic management (two missing values)	8.3	91.7	4
8. Managerial decision-making (two missing values)	10.3	89.7	7

Results in Table 3 indicated that the respondents’ required levels of knowledge regarding practice management tasks were high or very high for all eight business tasks. Respondents were aware of the high need for practice management knowledge. Respondents were of the opinion that they required the most knowledge about the practice management task of marketing (95.8%). Marketing is the creation of demand for a particular product or service by establishing public awareness (Taylor, 2015b; Traynor, 2006). Audiologists have to market their services and qualifications to the community to create greater awareness of the available services, but must also follow the ethical rules regarding advertising (HPCSA, 2008). This is especially applicable to private practitioners, where marketing is integral to their success (Taylor, 2015b). Therefore, the development of marketing skills should be as much a priority as the development of hearing evaluation skills (Kotler & Keller, 2009; Staab, 2008). Finance, accounting and other business functions will be irrelevant in the absence of a sufficient demand for products and services in order to make a profit (Kotler & Keller, 2009).

Legal and ethical issues (95.2%) were also rated highly. Finance (91.8%), accounting (91.7%) and strategic management (91.7%) were considered by the respondents to be equally important. An ability to understand the financial drivers of a successful practice is a fundamental and long-lasting skill set that will benefit any professional regardless of his or her work setting (Traynor, 2008). Respondents realised the importance of this by rating the required levels of knowledge highly. Organisational behaviour, managerial decision-making, operations and systems management were rated lower in terms of required knowledge.

### Differences between the existing levels of knowledge and the required levels of knowledge necessary to perform practice management tasks as perceived by the respondents

By combining the two tables, differences in knowledge can be highlighted. The difference between the required and the existing levels of knowledge levels is indicated in Table 4.

**TABLE 4:** Difference between respondents’ existing levels of knowledge and required knowledge necessary to perform practice management tasks (*n=*147).

Management task	Required knowledge (%)	Existing knowledge (%)	Difference (%)	Significance (p-value=0.05)	Ranking of differences
1. Accounting	91.8	37.4	54.4	0.000	2
2. Finance	91.8	42.9	48.9	0.000	4
3. Marketing	95.8	43.5	52.3	0.000	3
4. Legal and ethical issues	95.2	39.50	55.7	0.000	1
5. Organisational behaviour and human resources	89.1	44.9	44.2	0.000	7
6. Operations and systems management	89.7	56.2	33.5	0.000	8
7. Strategic management	91.7	43.5	48.2	0.000	6
8. Managerial decision-making	89.7	40.8	48.9	0.000	5

As indicated in Table 4 the difference in knowledge is the difference between the percentages of respondents who described their existing and required levels of knowledge as high or very high. This result was not obtained from the respondents directly but rather serves as an informative way to summarise the data and highlight differences in knowledge as previously done by Henson *et al*. (2006). As indicated in Table 4 the Z-proportional test revealed statistically significant differences between the required and existing levels of knowledge for all eight practice management tasks (*p* < 0.05). The results are in agreement with Henson *et al*. (2006), who also found a significant difference between required and existing levels of knowledge amongst respondents in the USA and Canada. The majority of the respondents (95.2%) in the current study were of the opinion that audiologists need high or very high levels of knowledge about legal and ethical issues to effectively manage audiology practices. In reality only 39.5% were of the opinion that they possessed high or very high knowledge about legal and ethical issues. The results were in agreement with Naudé and Bornman (2014), who found that despite the fact that knowledge of ethics in audiology grew between 1980 and 2010, retrospective analysis identified gaps in the current knowledge. This was the largest difference amongst the practice management tasks followed, again, by the difference in knowledge about accounting (54.4%) and marketing (52.3%). This disparity may partly be due to a lack of opportunity for audiologists to acquire fundamental knowledge of practice management. According to Traynor (2006) it is not surprising that audiologists perform outside their educated expertise in these areas.

### Comparison between existing and required levels of knowledge for public and private working environments

Table 5 indicates a comparison between the existing and required levels of knowledge amongst respondents working in public or private settings.

**TABLE 5:** Comparison of existing and required levels of knowledge between public (n=41) and private (n=92) working environments.

Management task	Existing knowledge private (%)	Existing knowledge public (%)	Difference between existing knowledge (%)	Required knowledge private (%)	Required knowledge public (%)	Difference between required knowledge (%)
1. Accounting	42.2	31.7	10.5	90.1	95.0	−4.9
2. Finance	48.9	31.7	17.2	91.3	90.2	1.1
3. Marketing	52.2	22.0	30.2	97.8	90.0	7.8
4. Legal and ethical issues	41.3	29.3	12.0	95.7	95.1	0.6
5. Organisational behaviour and human resources	50.0	31.7	18.3	91.3	85.4	5.9
6. Operations and systems management	68.1	34.1	31.0	91.2	85.4	5.8
7. Strategic management	48.9	31.7	17.2	90.0	92.7	−2.7
8. Managerial decision-making	40.2	41.5	-1.3	89.0	87.5	1.5

According to Table 5, there were small differences between existing knowledge in private and public settings. The Z-proportional test however, revealed statistically significant differences between existing levels of knowledge regarding marketing (*p* = 0.001), organisational behaviour and human resources (*p* = 0.049) and operations and systems management (*p* = 0.000). Respondents working in a private setting had a higher existing knowledge regarding these three tasks. Respondents working in a private setting may gain more experience regarding marketing as they have to actively market their practices. It is possible that respondents working in the public sector might be overburdened by the demand for their services and therefore have limited knowledge regarding marketing. Respondents working in the private sector are solely responsible for organisational behaviour and human resources. In a public setting these responsibilities are handled collectively. The Z-proportional test revealed no significant differences between respondents working in a public and/or private context regarding their required levels of knowledge in all eight of the practice management tasks. The results indicated that there is a great need for knowledge regarding practice management irrespective of the working environment. Respondents work in a variety of employment contexts throughout their careers with various common traits; for example, they all have to conform to legal and ethical constraints. Therefore, the required level of knowledge regarding legal and ethical issues is high, regardless of the employment context.

### Strengths, management challenges and training needs as perceived by respondents

Respondents indicated in the text data section that their biggest strengths were patient satisfaction, successful marketing, and starting their own practices. Respondents indicated that a lack of training, knowledge, experience and sufficient finances were their biggest challenges in practice management in South Africa. In the open comments section of the survey, one respondent stated that ‘a lack of knowledge and education before starting your own private practice is the biggest challenge in practice management in South Africa’. The majority of respondents indicated that training is required to overcome these challenges.

### Recommendations regarding audiology practice management training

According to Table 6, the majority of respondents (80.8%) believed that there is a need for practice management and that such training should be offered at an undergraduate level. Taking into consideration that audiologists working in both public and private settings had a low existing level of knowledge, training at an undergraduate level would be ideal. In reality, just under a quarter (22.7%) of the respondents indicated that it was presented as an undergraduate module, emphasising the need for change regarding practice management training. Continuing professional development (CPD) activities were also rated highly (57.7%). For audiologists to maintain their registration with the HPCSA they have to obtain 60 CPD points in a two year cycle (HPCSA, 2011). Therefore, CPD activities will be a good means to address the need for practice management training and acquiring CPD points.

**TABLE 6:** Summary of training recommendations (n=147).

Area	Top three recommendations of respondents			Options not favoured
		Recommendation 1	Recommendation 2		Recommendation 3	
When training should be offered	Undergraduate level (80.8%)	Continuing professional development (57.7%)	When entering private practice (34.6%)	Postgraduate level (28.9%)
Who should co-ordinate the training	Individuals with practice management experience (59.6%)	Department of Audiology at universities (59.0%)	SAAA (52.6%)	Entrepreneurs (7.05%)
Format of training	Short course (45.7%)	On-the-job training (43.1%)	Continuing professional development activities (23.4%)	Distance learning programme (4.8%)
Evaluation methods	Assignments throughout the course (60%)	Practical business project (32.9%)	Written business plan (28.4%)	Written exam and oral presentations (14.2%)
Topics for training	Marketing (93.5%)	Legal and ethical issues (91.5%)	Customer Service (91.5%)	Operations and systems management (73.2%)

When asked who should co-ordinate practice management training, individuals with practice management experience (59.6%), and departments of audiology at universities were rated highly. The latter indicated that respondents wanted to learn from lecturers with experience in the field, and that universities are held in high regard by the respondents. According to Fasokun, Katahoire and Akpovire (2005), experience is regarded as more important than knowledge amongst adults in South Africa, therefore respondents may have rated individuals with experience highly. Respondents recommended that practice management training should be presented as a short course (45.7%), or as on-the-job training (43.1%), but distance learning was not favoured (4.82%). Since respondents were mostly young female professionals in their thirties, employed full-time in private practices (Table 1) with little spare time, having to balance both career and family life they may have preferred short courses or in-service training. According to Fasokun *et al*. (2005) adult learners are physically, psychologically and culturally different from young learners. As a result of differences, adults apply habitual styles when learning. Owing to their individual needs, adult learners may easily feel left out of learning activities, and this may be why distance learning was not favoured by respondents (Baloglu, 2007; Fasokun *et al*., 2005). The majority of respondents (60%) preferred assignments throughout the course. According to Gibbs and Simpson (2004), adult learners consider coursework to be fairer than exams and measure a greater range of abilities. The quality of learning has also been shown to be higher in assignment-based courses (Gibbs & Simpson, 2004).

Marketing was rated as the most important topic to be included in training (93.5%), which is in agreement with the required levels of knowledge as indicated by the respondents in Table 3. Respondents have to promote their private practices, since these are essentially small businesses. According to Staab (2008), audiologists must understand the fundamental principles of marketing and have basic marketing skills. Great emphasis was placed on basic marketing skills as marketing the profession is also important for the future of audiology. Marketing was closely followed by legal and ethical issues (91.5%). Ethical considerations should go hand in hand with promoting a practice (Solodar & Williams, 2007). Audiologists have a professional code of ethics and standards as well as guidelines for good practice (HPCSA, 2008; SASLHA, 2011) that ensures high ethical standards and which provide the foundation for good customer service. According to Taylor (2015a), a trusting relationship with the audiologist is rated highly by patients. For this reason audiologists inherently place a large emphasis on legal and ethical issues.

## Conclusion

This study found significant differences between respondents’ self-perceived existing and required knowledge in all eight practice management tasks. Legal and ethical issues, as well as marketing and accounting, revealed the biggest differences. Respondents recognised that they need significant expansion of their practice management knowledge, skills and attitudes in order to be successful irrespective of their work context. The success of private audiology practices is also important for the future engagement between private and public sectors when the National Health Insurance system is implemented. To address these needs audiology programmes should incorporate aspects of all eight practice management areas that compose what is considered the most important body of knowledge for practice management (Henson *et al*., 2006; Hosford-Dunn *et al*., 2008). According to Henson *et al*. (2006), there are many options for audiologists in the USA and Canada seeking additional practice management education. This may include web-based learning, professional association conventions, continuing professional education activities, manufacturer support, mentoring, books and educational opportunities outside those tailored to the profession. Despite the assistance provided by these individual opportunities, Simpson (2011) states that one of the most prevalent means of business education for audiologists remains that of “trial and error”. According to Bakker (2008), audiologists receive excellent clinical training, but limited or no formal preparation for the challenges that the management of a private practice brings. Audiology practice management is a specialised field as audiologists face unique challenges such as marketing restrictions stipulated by the HPCSA (2008). There are several alternatives that may address this need as perceived by the respondents, such as future research into the content and methods taught in undergraduate training programmes in South Africa to make specific recommendations for incorporating additional practice management training, as recommended by respondents. According to Henson *et al*. (2008) giving up clinical modules to practice management modules or extending programme durations will be difficult as the focus of most university programmes internationally is on clinical training, as this is the core professional function. Therefore, postgraduate training, continued professional education and short courses as recommended by the respondents, can be considered. Henson *et al*. (2008) recommended an industry-wide effort to develop and manage a practice management education programme designed specifically for healthcare professionals. This effort could be led by a national or international association and developed at universities that offer audiology programmes (Henson *et al*., 2008). Taking into consideration what audiologists know and what they should know and using their recommendations to make improvements, practice management training has the potential to enhance all aspects of the profession, improve service delivery, empower practitioners, create awareness of the profession and increase satisfaction of both providers and patients (Hosford-Dunn *et al*., 2008).

A limitation of the current study is the fact that the results reported on are derived from a small sample of audiologists and dually qualified speech-language therapists and audiologists in South Africa. The response rate was below the desirable rate described in the literature (Maxwell & Satake, 2006). As a result, the findings may be biased and cannot be generalised to the greater population of audiologists. Despite these limitations the data was stable as similar findings were reported by other studies (Bakker, 2008; Henson *et al*., 2006; Wemmer, 2007), and significant conclusions could be drawn about what respondents know and what they should know. Recommendations for further studies include that the same study be conducted with speech-language therapists. Most of the respondents (65.4%) were dually qualified as speech-language therapists and audiologists. Therefore, some of the respondents might still practice as speech-language therapists as well; hence, it is assumed that speech-language therapists would have similar practice management training needs, although this should be investigated further in a separate study.
